# MTHFD2: A Retrospective and a Glance into the Future

**DOI:** 10.3390/ijms262211025

**Published:** 2025-11-14

**Authors:** Costas Koufaris, Vicky Nicolaidou

**Affiliations:** 1 Cyprus Cancer Research Institute, Nicosia 2109, Cyprus; 2 Department of Life Sciences, School of Life and Health Sciences, University of Nicosia, Nicosia 2417, Cyprus

**Keywords:** MTHFD2, one-carbon metabolism, retrospective

## Abstract

The 1985 confirmation by Mejia and MacKenzie of mammalian NAD-dependent methylenetetrahydrofolate dehydrogenase activity launched four decades of research on methylenetetrahydrofolate dehydrogenase (NAD^+^-dependent), methenyltetrahydrofolate cyclohydrolase 2 (MTHFD2). This review provides a retrospective on four decades of advancements on MTHFD2 that have revealed its key roles in the folate pathway, amino acid and redox homeostasis, and the metabolism of cancer and immune cells. We trace the initial biochemical characterization of the enzyme, highlight pivotal discoveries regarding MTHFD2’s metabolic and non-canonical roles, and discuss the current state of knowledge and future prospects.

## 1. Introduction

Folate refers to a large number of chemical species, consisting of a 2-amino-4-hydroxy-pteridine ring linked by a methylene (CH_2_) group to a p-aminobenzoyl moiety, with a further connection through an amide bond to the α-amino group of a monoglutamate or poly-γ-glutamate. Mammals, including humans, are unable to synthesize folate and thus require sufficient dietary intake to maintain its levels. In adults, folate deficiency causes anemia, whereas this deficiency during developmental stages may cause various birth defects, including different types of neural tube defects [1]. A central function of folate species is as carriers of one-carbon (1C) units, primarily derived from amino acids such as serine, glycine, and methionine, which can be attached to N5, N10 or both. The carrier of 1C units is usually the biologically active form of folate, tetrahydrofolate (THF) [2]. Folate derivatives then serve as key cofactors for a range of essential reactions. Specifically, methyl-THF (CH–THF) is a critical methyl donor for numerous methylation reactions, methylene-THF (CH_2_–THF) is required for the conversion of dUMP to dTMP—a rate-limiting step in DNA synthesis—and formyl-THF (CHO–THF) is utilized in the de novo synthesis of purines. Beyond these well-established functions in nucleic acid synthesis, a significant recent finding has elucidated the pathway’s crucial role in redox homeostasis. The conversion of CH_2_–THF to CHO–THF by the bi-functional methylenetetrahydrofolate dehydrogenase–methenyltetrahydrofolate cyclohydrolase enzyme generates a substantial amount of NADPH, a key cofactor that powers reductive biosynthesis and cellular antioxidant defences. Folate metabolism also plays a role in amino acid homeostasis, allowing the synthesis and conversion of serine, glycine, and methionine. Hence, high activity of the folate pathway is required to fuel the metabolic demands of rapidly proliferating cells, making it a prime target for therapeutic intervention [3,4,5,6].

**Figure 1:**
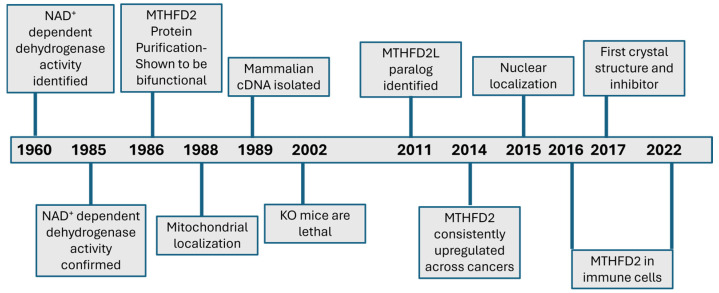
Timeline of major milestones and advancements for MTHFD2.

Folate and 1C metabolism (1CM) have been one of the earliest targets of anti-cancer therapies, all the way back to aminopterin in 1948 [7]. Beyond cancer, antifolates have also been useful as antimicrobial and immunosuppressant drugs [8]. It is no surprise, therefore, that considerable effort has been invested into elucidating the pathways, identifying the implicated enzymes, and characterizing their biochemical and cellular functions. Many key enzymes of the folate pathway were identified in the 1950s–60s, the “golden age” of enzymology [9]. These included the enzymes DHFR and TYMS, which are the targets of the methotrexate and 5-FU, respectively. In this review we are focusing on another enzyme of folate-dependent 1C metabolism, that is possibly in the midst of its own golden period, MTHFD2. This year marks the 40th year since the publication of the work by Mejia and MacKenzie confirming the presence of MTHFD2 enzyme primarily in transformed cells [10]. Since then, dedicated and often inspired commitment and efforts of researchers over decades have revealed a number of fascinating and unexpected observations regarding this enzyme (see Figure 1 for a timeline of major milestones). In this review, we will present a retrospective analysis on how knowledge on MTHFD2 has advanced to the present day, and a discussion of the current state and future prospects.

## 2. Phase I: Identification and Biochemical Characterization of MTHFD2

The first indication for the existence of an enzyme with the properties of MTHFD2 was in an early study by Scrimgeour and Huennekens reported in 1960 [11]. At that stage, it had already been established that mammalian tissues contained an NADP^+^-dependent methylenetetrahydrofolate dehydrogenase, which would later be determined to be MTHFD1. The authors were then inspired to examine whether additional NAD^+^-dependent enzyme(s) that could catalyze this reaction could also be identified. For this purpose, the isolated cell extracts from the then-available Ehrlich ascites tumour cells, a mouse undifferentiated carcinoma, were used. The cellular extracts were mixed with synthesized N5,N10-methylene FH4, and an assay for the dehydrogenase activity was performed. The authors were then able to confirm the presence of both NAD^+^- and NADP^+^-dependent enzymes in these crude extracts. They also noted that the NAD^+^-dependent enzyme only had a requirement for Mg^++^, an observation that would be noted in the future. And this is where things stood regarding the NAD^+^-dependent dehydrogenase for the next 25 years. Why was this finding then overlooked, if not forgotten? The technologies of the time did not allow the easy purification of the enzyme, let alone its cloning and production in large quantities. Its identification in only a single mouse cell type, its absence from the majority of tissues, and the presence of an alternative methylenetetrahydrofolate dehydrogenase in mammalian cells likely led researchers to consider it as a non-essential enzyme. Ironically, it would be its distinctive expression patterns that would attract considerable interest decades later.

By the mid-1980s, work by several groups, including Robert E. MacKenzie at McGill University, had firmly established that the NADP^+^-dependent enzyme was actually tri-functional, possessing dehydrogenase, cyclohydrolase and formyltetrahydrofolate synthetase activities [12]. The MacKenzie group then decided to revisit the unsubstantiated report of an NAD^+^-dependent dehydrogenase from the original 1960s publication. It is not clear to us what motivated this decision, inspired as it would eventually prove to be. Was that observation lingering in the mind of MacKenzie for a long time, or did he happen to serendipitously be led to the path of a new research direction? It would be reasonable to assume that Professor MacKenzie was inspired to look for the mammalian NAD^+^-dependent methylene-THF dehydrogenase by the reports of such enzymes in bacteria in the early 1980s. As part of his long-standing interest in multi-functional folate enzymes, he was aware of these reports, as they are included in his book chapter in *Folates and Pterins: Chemistry and Biochemistry of Folates Vol. 1* (1984) [13]. In support of this presumption, the first two references of the Mejia and MacKenzie (1985) manuscript are precisely that book chapter review followed by a 1984 paper describing a bacterial NAD^+^-dependent methylene-THF dehydrogenase [14].

Either way, the first step the researchers took was to reconfirm the NAD^+^-dependent dehydrogenase activity in Ehrlich ascites cells. Chromatography was then used to separate the NAD^+^ and NADP^+^ activities. Next, using biochemical assays, they confirmed the presence of NAD^+^ dehydrogenase activity in 21 different cell lines. Interestingly, in their examination of mouse tissue, NAD^+^ dehydrogenase activity was detected only in the bone marrow, thymus, and spleen. Activity was also detected in embryonic rat liver, but not in the adult organ. Finally, NAD^+^ dehydrogenase activity was increased compared to NADP^+^ in cells transformed by SV40 or EBV. Thus, it was demonstrated clearly for the first time that mammalian transformed cells possessed at least two distinct enzymes able to perform the dehydrogenase–cyclohydrolase reactions [10]. The MacKenzie group followed its 1985 work for the next two decades, using the tools and techniques available at the time for characterizing the newly discovered enzyme.

A year after their 1985 publication, the MacKenzie group reported the purification of the NAD^+^-dependent enzyme, and showed it to be bi-functional, exhibiting cyclohydrolase activities [15]. Additionally, its molecular weight was found to be considerably smaller in size than the NADP^+^-dependent dehydrogenase [15]. Their next big revelation was the discovery that the MTHFD1 and the MTHFD2 protein differed not only in their cofactor dependencies, kinetics, and being tri- or bi-functional but also in their subcellular localization. Specifically, the authors discovered that the NADP^+^-dependent enzyme was primarily in the cytosol, while the NAD^+^-dependent enzyme was primarily in the mitochondria [16]. Subsequent isolation of the mammalian cDNA of MTHFD2 found the presence of an N-terminal mitochondrial localization signal, adding further support for its mitochondrial localization [17]. The mitochondrial metabolic function of MTHFD2 was not clear at that time due to missing puzzle pieces that would be slowly identified. In an influential, and largely correct, as would be proven, review published in 1991, Appling had proposed the compartmentalization of folate-mediated 1C metabolism in eukaryotes [18]. However, MTHFD2 is bi-functional, so it could not complete the mitochondrial 1C metabolism like the tri-functional cytosolic MTHFD1. There are limitations to the ability of folate species to cross the mitochondrial membrane [19]. Monoglutamate folates cannot freely cross the mitochondrial membrane, but they can be transported into the mitochondrial matrix by the mitochondrial folate transporter. Polyglutamate folate is unable to cross the mitochondrial membrane. Once inside the mitochondria, the enzyme folylpolyglutamate synthetase (FPGS) converts monoglutamate folate to polyglutamate, but thereafter, it becomes trapped within the mitochondrial matrix. The answer to this puzzle was the subsequent identification of a mitochondrial uni-functional 1C-THF synthase [20,21]. Generation of purines from mitochondria was also blocked in *MTHFD2*^-/-^ fibroblasts [22]. Thus, MTHFD2 is part of a parallel mitochondrial 1C folate pathway. Another contribution of the MacKenzie group was the generation of *MTHFD2*^-/-^ mice, which were found to be embryonic lethal [23], demonstrating an essential embryonic function for this gene.

**Figure 2:**
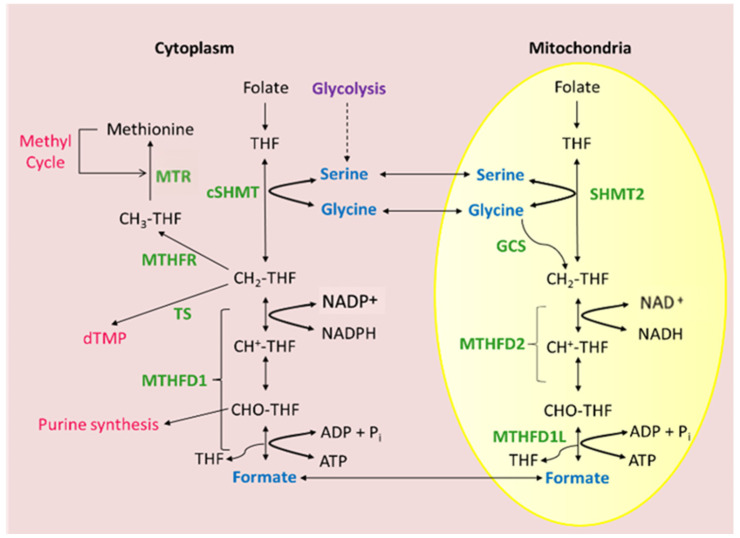
Current picture of MTHFD2 as mitochondrial NAD^+^-dependent methylenetetrahydrofolate dehydrogenase/cyclohydrolase. Mammalian folate-dependent 1CM is compartmentalized into cytosolic and mitochondrial compartments. Mitochondrial 1C flux secretes formate into the cytosol where it can be used to synthesize purines and thymidylate.

Collectively, much progress had been achieved from the initial observation of Scrimgeour and Huennekens in the 1960s, namely the characterization of the core properties of this enzyme: a nuclear-encoded mitochondrial NAD^+^-dependent enzyme with a requirement for inorganic phosphate and magnesium ions catalyzing two steps of 1C metabolism, the conversion of CH_2_-THF to CH^+^-THF and, subsequently, to CHO-THF (Figure 2). Nevertheless, many open questions remained. Why do mammalian cells possess two parallel folate 1C pathways, and what is the contribution of each? How is MTHFD2 expression regulated in cancer cells, and is it needed to drive their enhanced growth? Could effective inhibitors of MTHFD2 that can target this isozyme specifically be developed? To a large extent, these questions are still relevant today.

## 3. Phase II: MTHFD2 Emerges as a Promising Cancer Target

**Figure 3:**
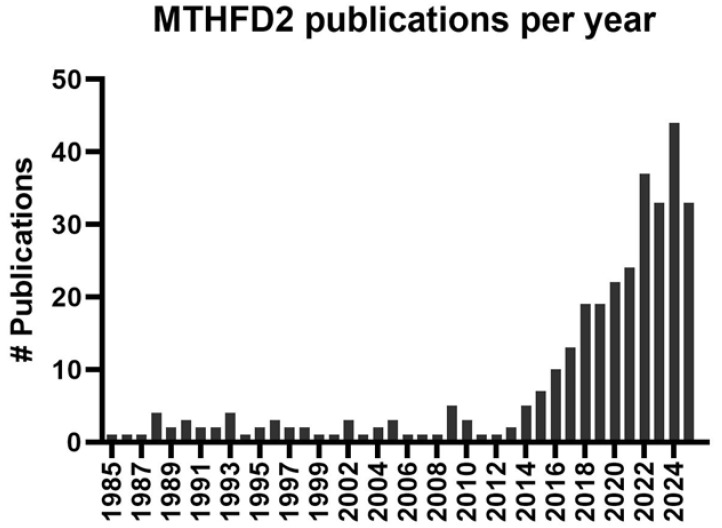
MTHFD2 publications per year. A PubMed search was performed using “mthfd2”, “nmdmc”, and “NAD^+^-dependent methylenetetrahydrofolate dehydrogenase” as keywords. The Y-axis depicts the number (#) of publications found in the PubMed database using these keywords per year.

Perhaps sadly, wider interest in MTHFD2 would not be achieved until the middle of the past decade, after the retirement of Professor RE MacKenzie, who had pioneered this topic and worked on it for two decades (Figure 3). This revival and greatly expanded interest was motivated largely by the work of Nilsson et al., 2014, which used more modern approaches and tools to highlight MTHFD2 as a highly promising target for cancer therapy [24]. Notably, this was a large untargeted meta-analysis examining 1454 metabolic enzymes across 1981 tumours and spanning 19 cancer types. A potentially important link between higher MTHFD2 expression and cancer was already suggested by the earliest work on this enzyme. The original Scrimgeour and Huennekens 1960 manuscript concludes with this off-hand observation “*In contrast to the TPN enzyme, the DPN enzyme cannot be detected in normal mouse liver, in the liver of mice bearing the ascites tumor or in chicken, rabbit, cod, salmon, and human liver*” (where TPN and DPN refer to the old nomenclature for NADP^+^ and NAD^+^, respectively) [11]. The potential connection of MTHFD2 with cancer was more strongly supported and explicitly stated in the 1985 Mejia and MacKenzie paper: “*From work presented here, it is clear that the functional role of NAD-dependent methylene-H4 folate dehydrogenase in development and transformation is an important question*” [10]. These studies, limited by the tools of their age, had only examined MTHFD2 in a few cell/tissue types. Nilsson et al., however, remarkably found that MTHFD2 was the consistently induced metabolic enzyme between tumours and normal tissues. Additionally, silencing of MTHFD2 using RNAi was lethal to cancer cell lines. Their analysis also suggested that MTHFD2 expression was lower in most types of mitotic non-transformed cells compared to transformed cells. Collectively, this paper painted the picture of MTHFD2 as a highly appealing novel cancer target, apparently being both required and restricted to transformed cells of several cancer types. Additionally, as a metabolic enzyme, its activity could potentially be readily druggable.

Cancer metabolism as a field has had a remarkable resurgence in the previous decade, with the renewed importance given to the Warburg effect and the reorganized metabolism of cancer cells [25,26]. Within this environment, MTHFD2 attracted considerable interest for the first time, and indeed, a large number of studies have confirmed the transcriptional upregulation of MTHFD2 across cancer types: breast [27,28], AML [29], glioblastoma [30], and kidney cancer [31], among others. The expression pattern of MTHFD2 and the detrimental effects of its targeting on cancer cells offered a strong rationale and motivation for the development of inhibitors against this enzyme. Indeed, such efforts are ongoing, as we will see below.

## 4. Phase III: Metabolic Compensation, Non-Canonical Functions, and Discovery of a Paralog

As is not atypical in science, the increased research and focus on MTHFD2 revealed additional levels of complexity to the initial understanding of how and where this enzyme functions. For MTHFD2, the new insights were (i) the metabolic compensation from the cytosolic pathway, (ii) the non-canonical functions of MTHFD2, and (iii) the identification of the MTHFD2L paralog. These new aspects of MTHFD2 are the focus of intense investigations and will greatly affect the design and development of therapeutic avenues based on the targeting of MTHFD2.

An interesting question raised from the initial recognition that eukaryotic cells possess two parallel folate-dependent 1C pathways was their relative contribution to the cellular 1C flux. The MacKenzie group had shown that *Mthfd2*^-/-^ fibroblasts could grow adequately in culture, despite being glycine auxotrophs [22]. Therefore, the cytosolic pathway is evidently sufficient to cover the metabolic demands of cells by itself in at least some circumstances. Why, then, did eukaryotic cells evolve this compartmentalization of 1C metabolism? Potential benefits could include greater regulatory flexibility, redundancy, and energetic advantages [32]. One possibility is that mitochondrial 1C metabolism is required to serve internal functions within the organelle that include N-formylmethionine pre-translational modifications of proteins, de novo thymidylate biosynthesis, and tRNA modifications [33]. Although these intra-organelle demands necessitate the existence of local 1C metabolism, the demands for 1C-derived units within the mitochondria are small compared to the total cellular demands. Consequently, the prominent flow that is observed through the mitochondrial branch is not justified. Ducker and Rabinowitz (2017) have proposed that the rationale for this compartmentalization relates to the common use of cofactors between metabolic pathways [3]. More specifically, they noted that 1C metabolism through MTHFD2 will result in the generation of NADH, which is otherwise primarily a product of TCA reactions. They then proposed that this could disrupt glucose fermentation in the cytosol that is balanced by NAD^+^ levels. Thus, separation of NAD^+^ usage from glycolysis and mitochondrial 1C metabolism can reduce undesired interactions between the two pathways.

An additional question that naturally emerges from the presence of two independent branches of eukaryotic 1C metabolism relates to the ability of the cytosolic branch to compensate for reduced mitochondrial flux, as can occur following the targeting of MTHFD2. As noted previously, *Mthfd2*^-/-^ murine fibroblast can grow normally in culture [22]. Ducker et al., 2016 used a combination of genetic manipulations with metabolomic profiling to carefully dissect the contribution of the parallel 1C folate pathways [34]. This study found that while cultured mammalian cell lines primarily relied on flux through the mitochondrial pathway, they could also rapidly compensate for disruption of the mitochondrial 1C pathway by increasing flux through the cytosolic pathway. Does targeting of MTHFD2, therefore, create metabolic deficiencies in cancer cells, or can suppression of the enzyme be compensated by the increased flux through the cytosolic pathway? The normal growth of murine *Mthfd2*^-/-^ fibroblasts and MTHFD2 KO cell lines as described by Ducker et al. would suggest that these cells possess no pronounced metabolic perturbations. However, a number of studies indicate that this is not necessarily the case. In a metabolomic investigation of breast cancer, MCF7 cells with stably suppressed MTHFD2 were found to have widespread effects on serine/glycine homeostasis, glycolysis, and TCA metabolism [35]. Pikman et al., 2016, working with acute myeloid leukemia (AML), also found that knockdown of MTHFD2 impacted cellular metabolism, with the depletion of TCA intermediates and increased glycine dependence [29]. A more recent study in AML using both RNAi and inhibitors showed that these resulted in thymine depletion and consequent DNA damage [36]. Flux through MTHFD2 is also a meaningful, alternative source of NADH to the TCA cycle [37]. Consequently, under some circumstances at least, it appears that MTHFD2 and mitochondrial 1C flux are important as a source of additional purines and antioxidants. Discrepancies between studies regarding the ability of cancer cells to rapidly metabolically compensate for targeting MTHFD2 could be due to methodological differences. For example, genomic deletion of MTHFD2 in cancer cell lines [34] appears to have been more tolerated than transient inhibitions using RNAi or chemical inhibitors. Cell culture conditions also tend to be more nutrient-rich compared to in vivo conditions of growing tumours and could thus be more dependent on mitochondrial 1C metabolism products. Clearly delineating the extent and conditions whereby the cytosolic pathway is unable to compensate for targeting of MTHFD2 is an urgent subject with direct impact on the anti-cancer potential of targeting this enzyme.

Another level of complexity would be added through the discovery that mammalian cells possess not only two parallel 1C pathways but also a mitochondrial isozyme of MTHFD2. This discovery was made by Dean Appling’s group at the University of Texas at Austin, motivated by the efforts to understand the functioning of mitochondrial 1C metabolism in adult, non-transformed mammalian cells with low or absent MTHFD2. Through a bioinformatic screening of the human genome, the authors identified a paralog of MTHFD2, termed MTHFDL, that was highly expressed across adult human tissues [38]. Subsequently, this group proceeded to perform a biochemical characterization of this isozyme and showed that it possessed both CH_2_-THF dehydrogenase and 5,10-methenyl-THF cyclohydrolase activities and can use either NAD^+^ or NADP^+^. Its mRNA levels were also found to be low in embryonic tissue but expressed throughout all adult tissues [39]. Subsequent investigations found that although MTHFD2L is also found in cancer cells, it is expressed at much lower levels than MTHFD2, does not show the same pattern of pronounced upregulation in cancer cells, and is additionally not responsive to growth factors [40]. MTHFD2L is thus likely a housekeeping gene allowing a baseline of mitochondrial 1C folate flux, with isozyme switching to MTHFD2 occurring in many cancers. Would it then be preferable to specifically target MTHFD2, which is strongly expressed in cancer cells, while sparing the MTHFD2L isozyme? In any case, such a strategy would be challenging due to the high homology of MTHFD2 and MTHFD2L [41]. Another possibility is that, besides increased cytosolic flux, cancer cells could also utilize enhanced MTHFD2L activity to compensate for the inhibition of MTHFD2. This could involve compensatory increases in the levels of this enzyme or enhanced activity. Currently, there is no evidence supporting this possibility, although a greater understanding of the regulation and function of the MTHFD2L isozyme would be insightful in the development of MTHFD2 anti-cancer inhibitors.

Finally, with hindsight, the main feature of MTHFD2 that was not anticipated by the early work of Professor RE MacKenzie was the possibility that it also possesses biological functions distinct from its metabolic functions in folate metabolism. Non-canonical functions of metabolic enzymes have been identified for a number of enzymes [42], with the first study supporting this possibility for MTHFD2 coming from Roland Nilsson’s group at Karolinka Institute. In their 2015 study, these researchers showed for the first time that MTHFD2 was also localized in the nucleus [43]. A first attempt to decipher potential non-canonical functions identified that MTHFD2 is strongly co-expressed and physically interacts with RNA-processing elements [44]. Non-enzymatic functions in DNA repair have also been reported by independent studies. In mouse stem cells, MTHFD2 was reported to interact with EXO1 [45]. Another study found that enzymatically inactive MTHFD2 promotes nonhomologous end-joining in response to DNA damage by interacting with PARP3 and enhancing its ribosylation [46]. More recently, MTHFD2 was shown to have a role in homologous DNA repair in response to DNA damage [47]. This raises the possibility that MTHFD2 induction in cancer cells could also be beneficial to cancer cells through its effect on DNA repair. Could targeting the non-canonical function of MTHFD2 be therefore therapeutically useful in cancers? Targeting these functions of MTHFD2 could involve distinct strategies from an emphasis on the suppression of its metabolic functions, for example, the targeting of protein interactions, rather than enzymatic activity.

Therefore, as more knowledge relating to MTHFD2 has accumulated, more questions regarding its potential as a cancer drug target and the optimal strategies to use towards this goal have arisen. Cancer cells display a certain degree of metabolic plasticity with enhanced cytosolic 1C flux, mitochondria also possess a paralog enzyme, and MTHFD2 has additional non-metabolic functions. Although these newer observations do not exclude this enzyme from being a promising anti-cancer target, they do highlight the need for further research into its biological functions and the metabolic and other consequences of its inhibition in cancer cells.

## 5. Challenges, Uncertainties, and Opportunities for the Development of MTHFD2 Clinical Inhibitors for Cancer Therapy

A key characteristic of effective drugs is that they possess wide therapeutic windows, i.e., effective doses that induce minimal toxicity in healthy tissues. The work by Nilsson et al. [24] and others highlighted MTHFD2 as possessing a particularly wide therapeutic window, with an isozyme switch potentially specific to cancer cells. This was then a strong motivation for research into inhibitors for this enzyme as a potential novel cancer therapeutic target. Indeed, multiple independent groups have reported the discovery and development of the first MTHFD2 inhibitors (Table 1).

The first substrate-based inhibitor of MTHFD2 identified was LY345899 [48]. Importantly, the IC50 values of LY345899 were determined as 663 nmol/L for MTHFD2 (*n* = 7) and 96 nmol/L for MTHFD1; consequently, this drug is a more potent inhibitor of the cytosolic pathway. Thus, beyond the blockage of cancer-enriched MTHFD2, this drug will also inhibit the essential MTHFD1. This drug was shown to repress tumour growth and metastasis against colorectal cancer cells [49]. In a small study of glioblastoma cell lines, LY345899 was shown to be effective against these cancer cells, and especially prominently in the absence of glutamine [50]. Toxicity to non-cancer cells due to the inhibition of MTHFD1 will, however, remain a major issue. Progress was achieved through the development of DS18561882, showing a greater selectivity towards MTHFD2 [48]. In the same study, they found that this inhibitor could block tumour growth in a mouse xenograft model. This drug was also shown to be effective in combination with pemetrexed [51], CHK1 inhibitors [52], and cisplatin [53]. However, subsequent studies have reported that it is not as strongly selective for the MTHFD2 isozyme as desired and affects non-cancer cells [36]. There are ongoing reports of efforts by independent groups to develop more selective and potent MTHFD2 inhibitors [54,55].

An alternative approach towards targeting the MTHFD2 activity in cancer cells was inadvertently highlighted through the discovery of the TH9619 drug. This drug was developed using a substrate-guided lead optimization strategy with LY345899 as the foundation and led to the development of the more potent TH9619 [53]. Initial characterization for this drug found that it was not selective of MTHFD enzymes but was highly active against cancer cells [53]. Interestingly, a subsequent characterization found that this drug is unable to cross the mitochondrial membrane and can therefore only target MTHFD1 and the nuclear MTHFD2. This mechanism of action can be lethal to cells due to the accumulation of mitochondrial-released formate to the cytosol, leading to depletion of thymidylate synthesis [56]. This “folate-trapping” is thus an independent strategy for targeting cancer cells based on their increased mitochondrial 1C flux.

A number of issues and strategies, however, still need to be addressed and clarified for targeting MTHFD2 in cancer. The first question is why cancer cells ultimately upregulate MTHFD2, and how it contributes to the growth and survival of cancer cells. The answer to this question was initially suggested by Professor MacKenzie in 1985 to be the generation of additional purines required for DNA synthesis of proliferating cells. The rapid compensatory switching to cytosolic 1CM and the non-canonical functions of MTHFD2, however, can complicate this interpretation. Do the more nutrient-depleted in vivo conditions potentially require flux through the mitochondrial pathway? Is targeting of both the cytosolic/mitochondrial pathways needed to achieve anti-cancer efficacy, and what would be the non-target toxicity of this strategy? Efforts to target MTHFD2 have so far focused on the enzymatic functions of MTHFD2, but as we have discussed, there is accumulating evidence for its nuclear functions in DNA repair. Investigation of the effects of selectively targeting the nuclear functions of MTHFD2 could indicate whether distinct targeting strategies against this function of protein are needed, e.g., targeting protein–protein interactions. Finally, an additional complication for cancer therapy is the potential detrimental effect of MTHFD2 on the immune system, as we will see in Section 6, that could counter the benefits of targeting this enzyme in cancer cells.

## 6. The Emerging Role of MTHFD2 in Immune Cells

The emphasis on the oncogenic role of MTHFD2 and its potential as a novel drug target has inadvertently led to reduced attention given to the emerging recognition of this enzyme also having important function in non-cancer cells, especially in the immune system. A potential link between MTHFD2 and the immune system was already suggested in the 1985 paper by Mejia and MacKenzie who had reported the detection of the NAD^+^-dependent methylenetetrahydrofolate dehydrogenase activity, not only in tumour cells but also in the bone marrow, thymus, spleen, that is, organs relating to the immune system. Later, it was shown that MTHFD2 KO mice presented with a failure of the liver tissue to support hematopoiesis, in contrast to mice lacking the closely related pathway genes MTHFD1L and AMT. *Mthfd2*^-/-^ mice are also smaller and paler than their WT littermates and lack normal liver redness [23]. Preliminary evidence that MTHFD2 is transcriptionally induced in activated lymphocytes, unlike other proliferating cell types, were also reported in the studies by Nilsson [24,43].

The first comprehensive study that definitively showed the role of 1C metabolism in T cells came from the Haigis Lab at Harvard University [57]. Through an elegantly designed approach employing computational, in vitro and in vivo methods, it was shown that naive CD4^+^ T cell activation induces a programme of mitochondrial biogenesis and proteome remodelling. This distinct metabolic signature revealed 1C metabolism as a highly induced pathway that is crucial for T cell activation and survival. Some years afterwards, Sugiura et al., 2022 published their study that specifically highlighted the role of MTHFD2 in CD4^+^ T cells [58]. The authors of the study reported that MTHFD2 is a critical metabolic checkpoint for Th17/Treg phenotypes, skewing the balance towards the pro-inflammatory Th17. Moreover, they found that MTHFD2 is upregulated consistently in individuals with inflammatory disorders and in vivo targeting of the enzyme protected against multiple inflammatory disease models. A recent preprint has also reported a crucial role for MTHFD2 in CD8^+^ T cells and that targeting it was effective in a model of type I diabetes [59]. Beyond T cells, MTHFD2 has also been reported to be important for the expansion and function of B cells. It was first reported that MTHFD2 was among the induced proteins in EBV-infected B cells that proceed to hyperproliferate. This was interpreted as contributing to the survival of B cells by generating NADPH [60]. The importance of MTHFD2 in nucleotide synthesis and redox balance was also recently reported in germinal centre B cells, whereby repression of the enzyme reduced growth and antibody production [61]. Activated lymphocytes are among the fastest proliferating mammalian cells, and increased mitochondrial flux involving MTHFD2 appears to be important for sustaining their growth and withstanding associated stresses.

Beyond lymphocytes, MTHFD2 was shown to be important in the monocyte–macrophage lineage. In macrophages, it was interestingly revealed that MTHFD2 affected polarization through non-metabolic functions, specifically the direct interaction and repression of PTEN [62]. A more recent paper reported that MTHFD2 restrained IKKα/β-NF-κB activation and macrophage inflammatory phenotype by scavenging reactive oxygen species through the generation of NADPH [63]. Thus, MTHFD2 appears to have canonical functions in normal macrophages as well.

It is evident that a lot of progress has been achieved since the earliest use of antifolates for their anti-inflammatory properties and immunosuppressive effects, such as methotrexate, in the 1980s and are still in use in autoimmune and inflammatory settings. Targeting 1C metabolism through MTHFD2 could offer an alternative, novel and more targeted approach for clinically beneficial immunomodulation.

## 7. Conclusions

The long journey to the isolation and characterization of MTHFD2 began with the identification by Scrimgeour and Huennekens in 1960 of a distinctive enzymatic activity (NAD^+^-dependent methylenetetrahydrofolate dehydrogenase activity) in Ehrlich ascite tumour cells. At that point, gel electrophoresis had not been developed yet, so its characterization was necessarily crude. A generation later, the MacKenzie group determined and defined key characteristics of this enzyme: its bi-functional nature, localization in mitochondria, and its elevated expression in embryonic and cancer tissues. It would take another generation before MTHFD2 became the focus of research by a substantial number of scientists. We are now perhaps in the midst of the “golden age” of MTHFD2 research.

In the case of cancer, MTHFD2 holds potential, particularly for precision medicine, combination therapies, and biomarker development. The unique expression pattern of MTHFD2—markedly upregulated in several tumour types but considerably limited in most healthy adult tissues—positions it as a key candidate for precision medicine strategies. Additionally, the centrality of mitochondrial 1C metabolism to nucleotide synthesis, redox homeostasis, and of MTHFD2 non-enzymatically affecting DNA repair, opens the door to synergistic combination therapy strategies. It is a rational expectation that inhibition of MTHFD2 can enhance metabolic stresses associated with existing chemotherapeutics such as antifolates, especially in cancers resistant to conventional therapies. Key next steps towards this goal will include the development and refinement of appropriate drugs and strategies. In terms of biomarker usage, MTHFD2 is highly enriched in cancers and has been associated with disease prognosis and resistance to therapy [28,64,65,66,67]. This makes MTHFD2 a promising candidate as a biomarker for both disease diagnosis and the stratification of patient response to therapy. Both mRNA and protein have shown potential for this purpose in the examination of transcriptomic datasets and of tissue biopsies. The performance of larger retrospective and eventually prospective human cancer studies is thus justified.

Despite the initial emphasis on MTHFD2 as a gene induced in cancers, all the way back to the Mejia and MacKenzie 1985 article, the targeting of this enzyme could hold as much or even greater potential as an immunomodulatory strategy. Antifolates have been in use for suppression of the immune system; however, these have a much wider expression and likely off-target toxicity than MTHFD2. Notably, in vitro formate can rescue the effects of blocking the mitochondrial 1C pathway in T cells, suggesting that its role in these cells is principally metabolic [57,58]. There is then great potential for MTHFD2 as an immune target, building upon inhibitors already under development as anti-cancer molecules.

In the coming decades, it will be determined whether MTHFD2 can have clinical translational benefits similar to, or potentially greater than, other enzymes of the folate cycle.

## Figures and Tables

**Table 1:** Characterization of existing MTHFD2 Inhibitors.

Molecule Name	Mode of Action	Comments
LY345899	Substrate-based binding	Folate analogue; not selective for MTHFD2; effective against colorectal and glioblastoma cells
DS44960156, DS18561882	Substrate-based binding	Developed on a tricyclic coumarin scaffold; more selective for MTHFD2
Xanthine derivatives	Allosteric binding	Not selective for MTHFD2; low potency
Carolacton	Non-substrate binding	Natural product; not selective for MTHFD2; effective against colorectal cancer
Diaminopyrimidine Derivatives	Substrate-based binding	Selectivity for MTHFD2 compared to MTHFD1; AML mutant FLT3 cells particularly sensitive
TH9619	Substrate-based binding	Inhibits MTHFD enzymes non-selectively in assays; in cells, specifically targets MTHFD1 and nuclear MTHFD2

## Data Availability

No new data were created or analyzed in this study. Data sharing is not applicable to this article.
